# SARS-CoV-2 Bottlenecks and Tissue-Specific Adaptation in the Central Nervous System

**DOI:** 10.21203/rs.3.rs-3220157/v1

**Published:** 2023-09-11

**Authors:** Justin Richner, Jacob Class, Lacy Simons, Ramon Lorenzo-Redondo, Laura Cooper, Tanushree Dangi, Pablo Penaloza-MacMaster, Egon Ozer, Lijun Rong, Judd Hultquist

**Affiliations:** University of Illinois at Chicago; University of Illinois at Chicago; Northwestern University; Northwestern University; University of Illinois at Chicago; Northwestern University; Northwestern University; Northwestern University; Department of Microbiology and Immunology, College of Medicine, University of Illinois at Chicago; Northwestern University

**Keywords:** SARS-CoV-2, Viral Evolution, COVID-19, NeuroCOVID, Furin Cleavage Site (FCS), Central Nervous System (CNS)

## Abstract

Severe COVID-19 and post-acute sequelae of SARS-CoV-2 infection are associated with neurological complications that may be linked to direct infection of the central nervous system (CNS), but the selective pressures ruling neuroinvasion are poorly defined. Here, we assessed SARS-CoV-2 evolution in the lung versus CNS of infected mice. Higher levels of viral diversity were observed in the CNS than the lung after intranasal challenge with a high frequency of mutations in the Spike furin cleavage site (FCS). Deletion of the FCS significantly attenuated virulence after intranasal challenge, with lower viral titers and decreased morbidity compared to the wild-type virus. Intracranial inoculation of the FCS-deleted virus, however, was sufficient to restore virulence. After intracranial inoculation, both viruses established infection in the lung, but this required reversion of the FCS deletion. Cumulatively, these data suggest a critical role for the FCS in determining SARS-CoV-2 tropism and compartmentalization with possible implications for the treatment of neuroinvasive COVID-19.

## INTRODUCTION

SARS-CoV-2 is an enveloped, positive-sense, single-stranded RNA virus and the causative agent of COVID-19. The virus has caused more than 6.9 million people deaths over the last three years. Eleven different WHO approved vaccines have been developed, significantly reducing global hospitalizations and deaths^1^. The respiratory system is the primary location of SARS-CoV-2 infection where the virus replicates in lung epithelial cells^2^. SARS-CoV-2 can also disseminate to distal tissues including the heart, gastrointestinal tract, and the central nervous system (CNS)^3,4^. COVID-19 is associated with a number of extrapulmonary pathologies including neurological post-acute sequelae (Long-COVID or neuro-PASC), acute kidney injury, gastrointestinal distress, myocarditis, thromboembolism, and acro-cutaneous lesions^5^. The viral and host factors which contribute to these extrapulmonary pathologies are not well understood.

SARS-CoV-2 entry into a host cell is mediated by the viral spike glycoprotein (S). S is produced as a full-length immature form before it undergoes proteolytic cleavage and maturation. S is cleaved into the S1/S2 subunits by furin proteases during viral egress from the host cell at the furin cleavage site (FCS; 681-PRRAR-685)^6^. The receptor binding domain (RBD) within S1 binds to the angiotensin converting enzyme 2 (ACE2) on the surface of susceptible host cells and undergoes a conformational shift. Canonically, this shift exposes the S2/S2-prime cleavage site, which is cleaved by a surface bound protease, TMPRSS2. S2/S2-prime cleavage exposes the fusion peptide (FP) domain of S2, which results in detachment of the S1 domain^7^. The exposed FP inserts itself into the plasma membrane of the cell, before a conformational change of S2 initiates membrane fusion and release of the genome into the cytoplasm^8^. Alternatively, SARS-CoV-2 can enter the cell through the endosomal entry pathway following ACE2 receptor binding. Within the maturing endosome, the cysteine proteases cathepsin B/L cleave the S protein at the S2/S2-prime site allowing for S1 release and membrane fusion.

The continued evolution of SARS-CoV-2 has resulted in the emergence and spread of new viral variants with enhanced fitness^9–13^. Variants associated with an increase in transmissibility, disease severity, or immune evasion are considered ‘variants of concern’ (VOCs)^14^, and most of the mutations responsible for these fitness benefits lie within the S protein. Mutations that alter FCS cleavage efficiency and stability of the S1/S2 interaction are commonly found in VOCs. One of the first mutations found to enhance SARS-CoV-2 transmissibility was S:D614G, which was found to enhance S1/S2 stability following furin cleavage and to promote sampling of the open confirmation, which exposes the RBD for ACE2 binding^15,16^. Subsequent mutations in the Alpha (S:P681H) and Delta (S:P681R) variants within the FCS were found to enhance furin cleavage and promote viral entry at the cell surface^17^. Deletion of the FCS in SARS-CoV-2 results in attenuated disease compared to ancestral virus in mouse and hamster models^18^. Proteolytic cleavage of the FCS removes the covalent bond between the S1 and S2 domains and can occur throughout various steps of the viral lifecycle, including maturation, egress, and cell entry^19^. It has been demonstrated that cleavage of the FCS results in altered cellular tropism, a high TMPRSS2 processing rate, and increased viral transmission^20^. However, the mechanism behind the altered viral dynamics in ΔFCS viral variants and how this mutation alters viral pathogenesis is unknown.

Animal models of SARS-CoV-2 can provide mechanistic insight into viral pathogenesis and viral evolution. One of the most commonly used models is the transgenic K18-hACE2 mouse which expresses human ACE2 on epithelial cells behind a cytokeratin-18 promotor^21^. SARS-CoV-2 replicates to high titers in the lung and brain of K18-hACE2 mice and leads to viral encephalitis^22,23^. Alternatively, mouse-adapted virus (MA10) can bind to murine ACE2 and infect wild-type mice. MA10 replicates primarily in the lung leading to respiratory pathology and enhanced disease in aged mice. Similar to circulating SARS-CoV-2 strains in humans, MA10 can reach distal sites including the brain^3, 24–26,^.

Here we asked if preexisting immunity shapes viral evolution and how SARS-CoV-2 evolves within different host tissues. Utilizing two different mouse models, we discovered increased viral diversity within the CNS regardless of vaccination status, suggesting that immune privileged sites such as the CNS may serve as a site of SARS-CoV-2 diversification. We also found that the FCS is under positive selective pressure in the respiratory tract and negative selective pressure in the CNS.

## RESULTS

### SARS-CoV-2 neuroinvasion selects for FCS mutations

**Figure 1:**
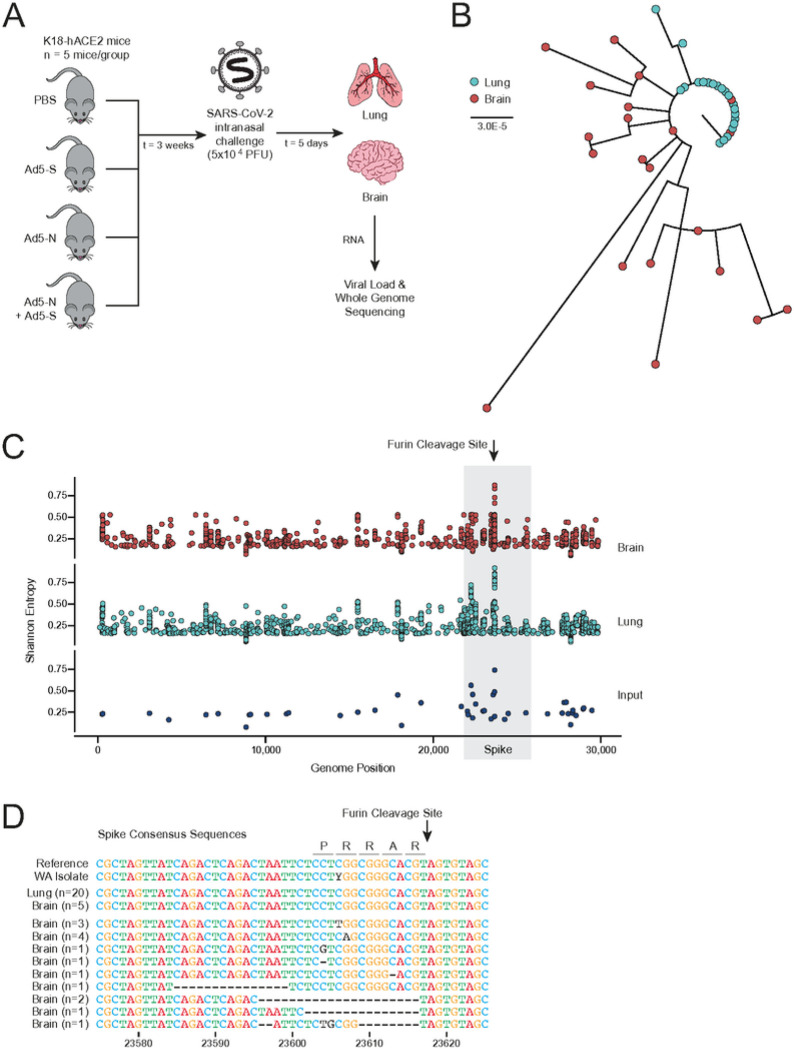
Viral RNA from the CNS has a higher degree of genetic diversity regardless of vaccination status. **a**, K18-hACE2 mice were vaccinated with Ad5 vaccines encoding spike, nucleocapsid, spike + nucleocapsid, or PBS as a negative control (n = 5 per group) three weeks before intranasal challenge with SARS-CoV-2. Brain and lung tissue were harvested 5 dpi. **b**, Phylogenetic tree of the consensus SARS-CoV-2 whole genome sequences (red tips = brain isolate, light blue tips = lung isolate). Branch length reflects nucleotide substitutions per site. **c**, Shannon entropy per position across the genome for the viral inoculate (n = 1, lower, dark blue), the lung isolates (n = 20, middle, light blue), and the brain isolates (n = 20, upper, red). The coding region for the spike protein is back shaded in gray with the FCS position labelled. **d**, Alignment of each consensus sequence across the Spike FCS region.

To better understand the determinants driving viral evolution and compartmentalization, we performed viral whole genome sequencing of SARS-CoV-2 in the lungs and brains of mice which had received different vaccine formulas. We hypothesized that vaccine-induced immunity against S and/or nucleocapsid (N) would drive viral evolution towards variants that escape antibody-dependent neutralization in a compartment-specific manner. To address this hypothesis, K18-hACE2 mice were intramuscularly vaccinated with adenovirus type-5 (Ad5) vector vaccines that encoded either the SARS-CoV-2 S open reading frame (Ad5-S) or the N open reading frame (Ad5-N) from the SARS-CoV-2 isolate USA-WA1/2020, similar to our previous studies^26–30^. Mice were vaccinated with either 1×10^9^ PFU of Ad5-S, 1×10^9^ PFU of Ad5-N, 1×10^9^ PFU of both Ad5-S and Ad5-N each, or with PBS as a control (n = 5 mice per condition). After three weeks, mice were challenged intranasally with 5×10^4^ plaque forming units (PFU) of USA-WA1/2020 and ultimately euthanized at 5-days post infection (dpi) (Fig. 1a). Total RNA from lung or brain homogenate was analyzed for the presence of viral RNA by quantitative reverse transcription PCR (qRT-PCR) and used for viral whole genome sequencing using an amplicon based approach as previously^31–33^.

Whole genome sequencing and phylogenetic analysis of the viral isolates from each condition revealed that, while the lung isolates were highly similar across all vaccination groups, the brain isolates were much more diverse (Fig. 1b). To better understand the distribution of these changes, Shannon entropy (a measure of viral diversity) was calculated at every position along the genome for the brain isolates, the lung isolates, and the USA-WA1/2020 input inoculate (Fig. 1c). While several positions varied within each compartment, there was a notable enrichment in diversity in S with the most diversity occurring in and around the FCS. Examining the consensus sequences of each isolate in this region, we found all 20 lung isolates had a majority consensus sequence that matched the reference with an intact FCS (Fig. 1d) regardless of vaccination. In stark contrast to this, 15 of the 20 brain isolates had a majority consensus sequence with a substitution mutation, frameshift, or deletion in or near the FCS.

**Figure 2:**
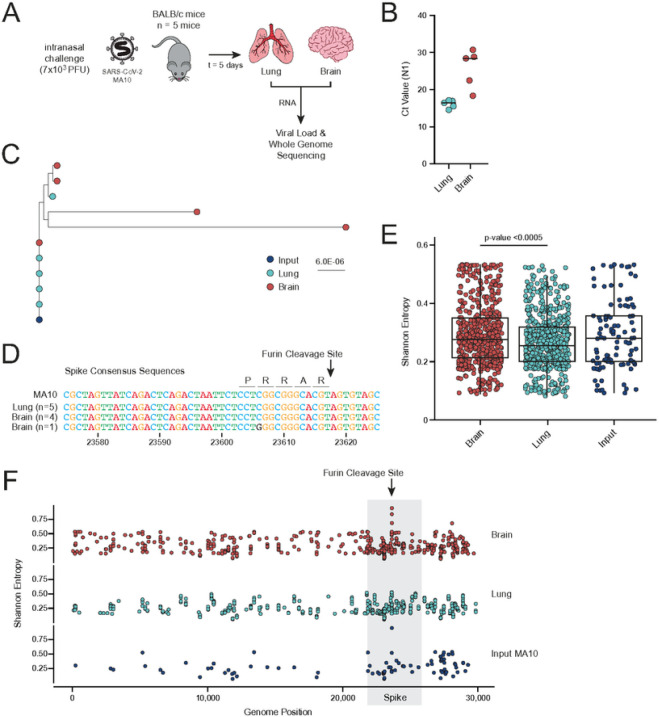
BALB/c mice infected with mouse-adapted SARS-CoV-2 have increased viral diversity in the CNS. **a,** BALB/c mice were intranasally challenged with mouse-adapted SARS-CoV-2 (n = 5 mice). Brain and lung tissue were harvested 5 dpi. (n=5). **b**, Ct values of the SARS-CoV-2 viral RNA (N1 target) isolated from the brain and lung of each mouse as quantified by RT-qPCR. The black line indicates the average Ct value per compartment. **c**,Phylogenetic tree of the consensus SARS-CoV-2 whole genome sequences (dark blue tip = inoculate, red tips = brain isolate, light blue tips = lung isolate). Branch length reflects nucleotide substitutions per site. **d**, Alignment of each consensus sequence across the Spike FCS region. **e**, Box plots of the Shannon entropy at each position across the genome for the brain isolates (n = 5, red), the lung isolates (n = 5, light blue), and the viral inoculate (n = 1, dark blue). Box plots represent the median and interquartile range. A log-transformed linear mixed effects model was used to test for significant differences in overall genetic entropy between tissues. **f**, Shannon entropy per position across the genome for the viral inoculate (n = 1, lower, dark blue), the lung isolates (n = 5, middle, light blue), and the brain isolates (n = 5, upper, red).

Notably, the parental isolate USA-WA1/2020 used in the above experiment was found to be polymorphic at position 23606 in the FCS. Sequencing of the USA-WA1/2020 inoculate yielded 47.9% reference sequence at that position while 52.1% of reads contained a C23606T (R682W) mutation that renders the FCS nonfunctional. Furthermore, SARS-CoV-2 is known to accumulate to very high titers in the tissues of K18-hACE2 mice, which may impact the selective pressures underlying compartmentalization. To test our findings in an alternate model, we intranasally challenged immunocompetent BALB/c mice with a mouse adapted strain of SARS-CoV-2 (MA10)^24^ and euthanized at 5 dpi (n = 5 mice, Fig. 2a). Total RNA was extracted from lung or brain homogenate to determine viral load and perform viral whole genome sequencing. While virus was detected in the brains of the MA10 infected BALB/c mice (Fig. 2b), titers were lower than in the USA-WA1/2020 infected K18-hACE2 mice. Whole genome sequencing and phylogenetic analysis of the viral isolates from each compartment revealed that the lung isolates again more closely matched the initial inoculate compared to the brain isolates (Fig. 2c). Two out of the five brain isolates showed substantial divergence from the input inoculate with one isolate’s majority consensus sequence harboring an R682G mutation in the FCS (Fig. 2d). Furthermore, Shannon entropy per position over the genome was significantly increased in the brain compared to the lung isolates (linear mixed effect model p < 0.0005, Fig. 2e). This diversity was again concentrated in the S open reading frame, with the highest peaks in entropy at sites in and around the FCS (Fig. 2f). Taken together, these data - in two independent mouse models with varying levels of CNS pathology and viral replication - suggest that neuroinvasion elicits a selective pressure for deletion or mutation of the FCS independent of prior immunity.

### FCS regulates the host cell entry pathway.

A previous study reported that deletion of the FCS in SARS-CoV-2 attenuates infection and reduces replication in the respiratory tract^34^, but it is not clear if this occurs in other organs of the body. The CNS has lower expression levels of ACE2 and TMPRSS2 compared to lung epithelial cells ^35–37^. The lack of TMPRSS2 in the CNS suggests that the virus must use an alternative entry pathway to target cells in the brain. The brain has high levels of cathepsin B and L expression and ACE2 can be found on epithelium, neurons, and vascular cells^38^. We thus hypothesized that the deletion of the FCS in the CNS is due to tissue specific selective pressure and utilization of the endosomal entry pathway.

**Figure 3:**
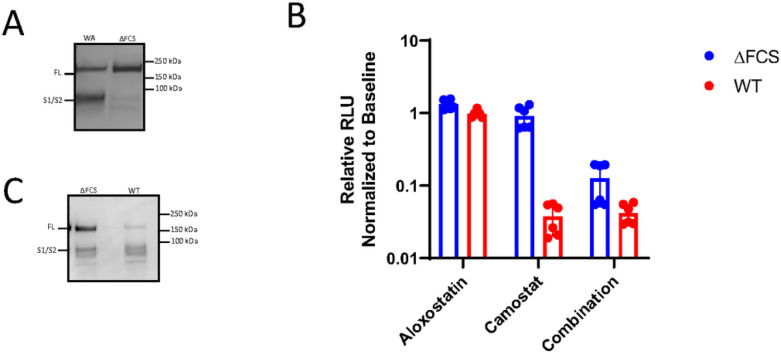
Mutation of the FCS blocks Spike S1/S2 cleavage and mitigates utilization of an alternative entry pathway. **a,** Pseudoviruses encoding for luciferase were generated with the WA-1 or ΔFCS deletion version of spike. Protein was isolated from pseudovirus cultures and subject to immunoblot staining against the spike protein. Shown is a representative blot of three independent western blots. **b**, Vero-E6 cells expressing hACE2 and TMPRSS2 were infected with WT or ΔFCS pseudotyped virus in the presence of presence of no drug, aloxostatin, camostat mesylate, or both. After 48 hours of infection, cells were lysed, and luciferase expression was quantified. Relative luciferase expression was normalized to untreated cells. Each condition was repeated three times. **c,** WT and ΔFCS virus were derived from infectious clones. Protein was isolated from viral stocks and subject to immunoblot staining against the spike protein. Shown is a representative blot of three independent western blots.

In order to evaluate the viral entry pathway of a virus lacking the FCS, we generated luciferase expressing pseudoviruses with an HIV backbone and SARS-CoV-2 S glycoprotein on the surface. The pseudovirus was generated with the S amino acid sequence from the parental WA-1 strain of SARS-CoV-2 or S with the amino acids 681-PRRA-684 deleted (ΔFCS). Loss of the FCS prevents furin-mediated processing of the S protein, resulting in higher levels of full-length S protein compared to the cleaved S1 and S2 domains as demonstrated by Western blot on the purified WA-1 and ΔFCS pseudoviruses (Fig. 3a).

To interrogate whether the ΔFCS pseudovirus exploits an alternative entry pathway, we transduced cells in the presence of TMPRSS2 and/or cathepsin B/L protease inhibitors. Expression of luciferase was used to determine transduction efficiency in VeroE6 cells overexpressing hACE2 and TMPRSS2. Cells were treated with 10μM of aloxostatin (E64d), which inhibit cathepsins, and/or 10μM of camostat mesylate, which inhibits TMPRSS2 activity. Luciferase expression remained unchanged with aloxostatin, indicating that both WA-1 and ΔFCS can enter through the canonical TMPRSS2 mediated entry (Fig. 3b). However camostat mesylate treatment caused a 26-fold decrease in WT pseudovirus entry while the ΔFCS mutant entry was unaffected. Combinatorial treatment with both aloxostatin and camostat mesylate lowered transduction of the ΔFCS pseudovirus. This data indicates that the ΔFCS mutant can efficiently utilize either the endosomal entry or TMRPSS2 mediated entry.

### ΔFCS virus is attenuated in the lung but not in the brain.

**Figure 4:**
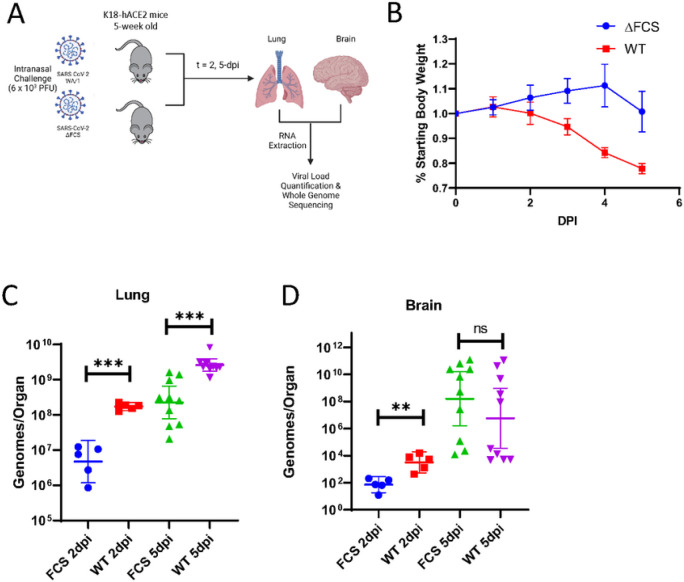
ΔFCS virus is attenuated after intranasal infection. **a,** K18-hACE2 mice were intranasally inoculated with WA-1 and ΔFCS stocks. Mice were intranasally challenged with 6×10^3^ PFU of wild-type (red/purple) or ΔFCS (blue/green) SARS-CoV-2 and **b**, evaluated for weight loss. (n = 15 for each virus). Viral genomes/organ are plotted from the **c**, lung and **d**, brain was isolated and quantified via RT-qPCR. At 2 days post-infection (dpi), n = 5 mice per virus were euthanized with remaining n = 10 euthanized at 5 dpi. Statistical significance is denoted by asterisks, as compared to WT virus (**p < 0.005; ***p < 0.0005; Unpaired T test).

Due to the increased prevalence of the ΔFCS mutant in the CNS, we hypothesized that the ΔFCS has a selective advantage within the CNS compared to the WA-1 virus. To address this hypothesis, we first obtained an infectious clone of the USA-WA1/2020 virus along with an infectious clone of USA-WA1/2020 with the ΔFCS mutation^18^. We confirmed that the S protein of the WT infectious clone and the ΔFCS infectious clone had differential cleavage of the S protein by western blot (Fig. 3c). After amplifying both viruses in VeroE6 cells expressing hACE2 and TMPRSS22, the WT viral stock contained 91.5% of reads with the WT FCS sequence and 8.5% of reads with mutations in the FCS region. The ΔFCS stock had 93.6% of reads with the predicted ΔPRRA deletion and 6.4% of reads with wild-type FCS. With these stocks we inoculated 5-week-old K18-hACE2 mice intranasally with 6×10^3^ PFU of either virus (Fig. 4a). As seen previously, the ΔFCS mutant caused significantly less weight loss in intranasally inoculated mice. At 4dpi, WT infected mice lost 17% body weight while ΔFCS infected mice gained ~ 5% body weight (Fig. 4b). Intranasally inoculated mice were euthanized at 2- and 5-dpi to provide a time course of viral dissemination during early infection. The ΔFCS mutant had an impaired growth rate compared to the WT virus with a 25-fold reduction in genomes/lung at 2-dpi (p-value = 0.0001) (Fig. 4c). This impaired growth phenotype was maintained through 5-dpi with 6-fold reduction in viral RNA titers in the lung of ΔFCS inoculated mice (p-value = 0.0002). We then asked if the deletion of the FCS provided improved entry into the CNS where it could then replicate. However, we found that at 2-dpi the ΔFCS had 58-fold lower genome/organ in the CNS compared to the WT virus (p-value = 0.0015). By 5 dpi the ΔFCS virus had slightly higher viral titers, although these differences were not significant (Fig. 4d). The delayed morbidity of the ΔFCS infected mice is likely due to the delayed viral kinetics in the respiratory system.

**Figure 5:**
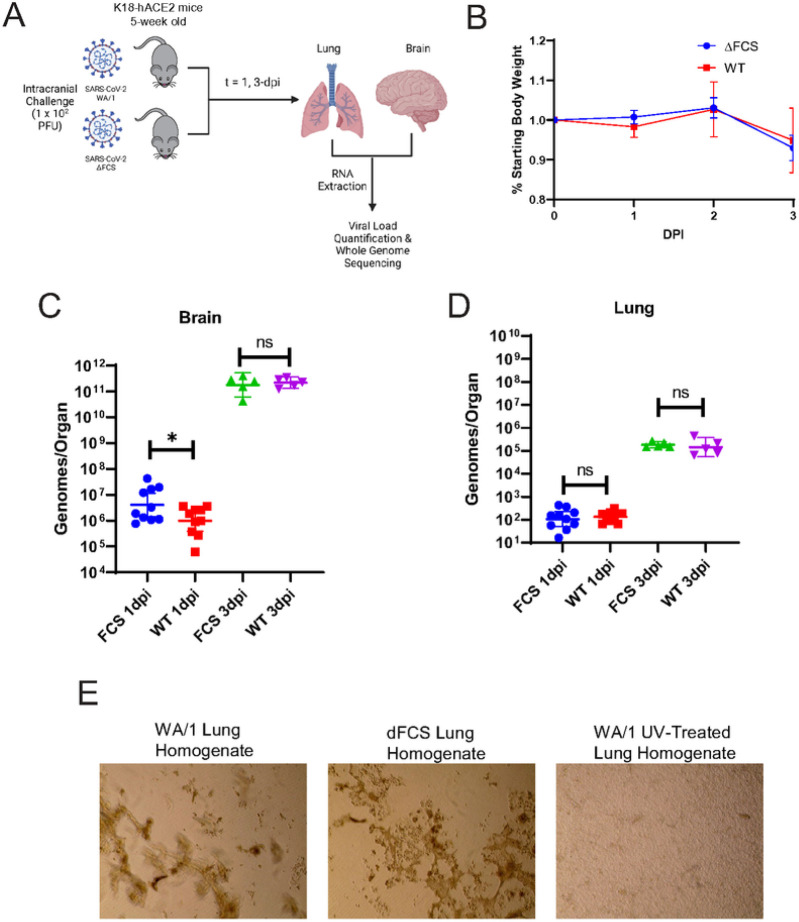
ΔFCS virus replicates faster in the CNS after intracranial inoculation. **a,** K18-hACE2 mice were intracranially challenged with 10^2^ PFU of wild-type (red/purple) or ΔFCS (blue/green) SARS-CoV-2. **b**, Mice were evaluated for weight loss (n = 15 for each virus). At 1 day post-infection (dpi), n = 10 mice per virus were euthanized with remaining n = 5 euthanized at 3 dpi. Viral RNA from the **c**, brain and **d**, lung was isolated and quantified via RT-qPCR. Viral genomes/organ are plotted. Statistical significance is denoted by asterisks, as compared to WT virus (*p < 0.05 Unpaired T test). **e**, Lung homogenate at 3dpi from WT or dFCS infected mice were inoculated onto a monolayer of Vero-E6 cells and incubated for three days. As a control, lung homogenate was UV treated to inactivate infectious virus in one group. Brightfield images of the monolayer is shown.

To assess CNS specific replication dynamics, we inoculated K18-hACE2 mice directly into the brain with 1×10^2^ PFU of WT or ΔFCS stocks (Fig. 5a). Following inoculation with either strain, mice reached humane endpoint criteria by 3 dpi; however, there was no clear difference in clinical symptoms or weight loss between the groups (Fig. 5b). We quantified viral titers in the brain and lung at 1 and 3 dpi. At 1 dpi, the ΔFCS mutant virus had increased viral loads in brain compared to the WT (4E6 vs. 9E5, p-value = 0.03), indicating that ΔFCS virus has an early growth advantage in the CNS (Fig. 5c). By 3 dpi, there was no difference in the viral loads in ΔFCS and WT infected mice. We also quantified viral genomes in the lungs of mice infected via the intracranial route to evaluate the possibility of leakage from the CNS into the respiratory tract. Low levels of viral genome copies in the lung were detected and these values increased over 1000-fold from day 1 to day 3, suggesting that viral dissemination can occur not only from the respiratory tract to the brain, but also from the brain to the lung (Fig. 5d). We could not detect infectious virus in the lung via plaque assays likely due to the limit of detection of this assay. However, *in vitro* infection of VeroE6 cells expressing ACE2 and TMPRSS2 with lung homogenate from intracranially infected mice resulted in cytopathic effect (Fig. 5e), indicating the presence of infectious virus. Collectively, these data demonstrate that, in contrast to the findings in the lung, the ΔFCS virus is not attenuated in the brain, and that virus originating from the brain can traffic back out to the respiratory tract.

**Figure 6:**
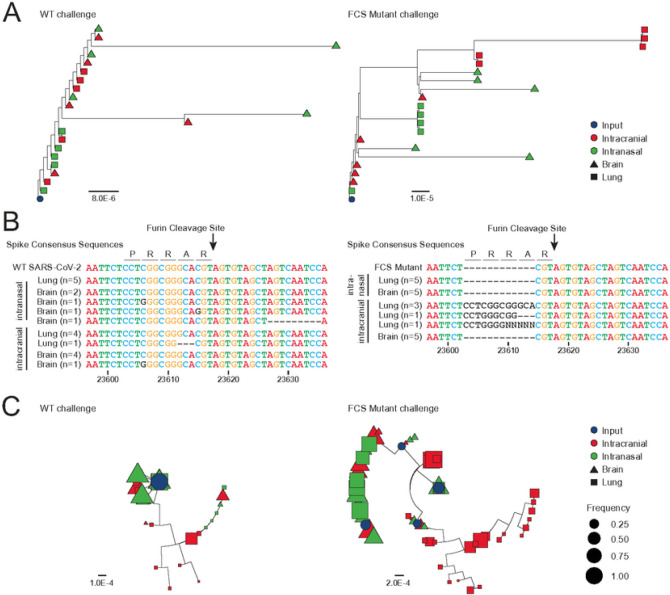
There is selective pressure for the deletion of the FCS in the CNS. **a,** Phylogenetic tree of the consensus SARS-CoV-2 whole genome sequences after intranasal or intracranial challenge with WT or ΔFCS virus (dark blue tip = inoculate, red tips = intracranial challenge, green tips = intranasal challenge, triangle = brain isolate, square = lung isolate). Branch length reflects nucleotide substitutions per site. **b,** Alignment of each consensus sequence across the Spike FCS region after challenge with the WT (left) or ΔFCS (right) virus. **c,**Phylogenetic tree of SARS-CoV-2 quasispecies after intranasal or intracranial challenge with WT (left) or ΔFCS virus (right) (dark blue tip = inoculate, red tips = intracranial challenge, green tips = intranasal challenge, triangle = brain isolate, square = lung isolate). Branch length reflects nucleotide substitutions per site and tip size reflects the frequency of a given subpopulation in an isolate.

To better understand the compartmental dynamics following intracranial or intranasal challenge, each isolate was again subject to viral whole genome sequencing and phylogenetic analysis. Mice infected intranasally with WT virus (left panels) displayed the previously observed pattern with the lung isolates (n = 5, green squares) grouping closely with the input inoculate (n = 1, blue circle) and with the brain isolates showing more divergence (n = 5, green triangles) (Fig. 6a). Following intranasal inoculation, the consensus sequence of the FCS in each lung isolate matched the reference whereas 3 of the 5 brain isolates had substitution mutations or deletions near or in the FCS (Fig. 6b). On the other hand, mice infected intracranially with WT virus had some lung isolates (n = 5, red squares) cluster more closely with the brain isolates (n = 5, red triangles) with more divergence from the input inoculate (Fig. 6a). Looking at the consensus sequences, 4 of the 5 brain and lung isolates maintained wild-type sequence across the FCS after intracranial inoculation, with one isolate in each compartment gaining a substitution or deletion mutation in that region (Fig. 6b). Quasispecies analysis confirmed that divergence of viral subpopulation after intracranial inoculation was maintained in the lung after trafficking (Fig. 6c).

Mice infected with the ΔFCS virus (right panels) showed a distinct pattern of viral evolution. Upon intranasal inoculation, some divergence is observed relative to the input inoculate (Fig. 6a), but all brain and lung isolates maintain the FCS deletion (n = 10, Fig. 6b). Upon intracranial inoculation, the FCS deletion is similarly conserved in all brain isolates (n = 5). However, the lung isolates of intracranially ΔFCS inoculated mice were highly divergent from the inoculating virus (red squares, Fig. 6a). Furthermore, each lung isolate was found to have partially (n = 2) or completely (n = 3) re-acquired the FCS sequence (Fig. 6b), which is similarly reflected in the quasispecies analysis (Fig. 6c). Taken together, these data suggest that trafficking of SARS-CoV-2 between the lung and CNS elicits a differential selective pressure for or against an intact FCS in Spike, respectively.

## DISCUSSION

Severe COVID-19 and post-acute sequelae of SARS-CoV-2 infection (PASC) have been associated with neurological symptoms including dysgeusia, anosmia, and brain fog, as well as more severe complications including delirium, strokes, and seizures^39,40^. This has been attributed to neuroinflammation as well as to direct infection of cells within the CNS^41,42^. Neuroinvasion by SARS-CoV-2 and other human coronaviruses has been previously reported by us and others, though the viral dynamics underlying this process is poorly understood^3,26, 43–45^. These data provide evidence that infection of the mouse CNS following intranasal challenge strongly selects for loss of the SARS-CoV-2 FCS in Spike. This same selection was not as strongly observed upon direct intracranial inoculation. Similarly, while an FCS-deleted virus was able to establish infection in the lung upon intranasal challenge, it required reversion of the FCS to populate the lung after intracranial inoculation. Taken together, this suggests that the selective pressure at the FCS is driven by a step in viral trafficking between the lung and the CNS.

Similar to our findings, the absence of the FCS in other coronaviruses has been associated with increased CNS tropism. For example, the human alphacoronavirus OC-43 naturally lacks an FCS, and when a neuroinvasive strain of OC-43 gained an FCS, dissemination into the CNS was decreased^46^. We would hypothesize that deletion of the FCS is selected for because the target cell type required for neuroinvasion has low TMPRSS2 expression, therefore putting pressure on the virus to favor an endosomal-mediated entry pathway. However, it remains unclear if this selective pressure is driven by tropism of a specific trafficked cell type or by population bottlenecking that amplifies selective pressure within a compartment. On one hand, inter-compartment trafficking may be mediated by a specific immune cell type that elicits a tropism-specific selective pressure, but the selection for an FCS when trafficking to the lung and for loss of an FCS when trafficking to the brain would require the involvement of different cellular intermediates dependent on directionality. On the other hand, population bottlenecking at the time of seeding may better amplify selective pressures within compartments that might not be observed upon direct inoculation due to sufficient initial challenge to overcome these barriers.

Several viruses are known to acquire compartment-specific adaptations that improve fitness in a given anatomical site, including Human Immunodeficiency Virus (HIV)^47^ and other coronaviruses. For example, murine hepatitis virus predominantly replicates within the liver, but mutations within the S protein can alter cellular tropism and cause neurovirulence. Similar to the data presented here, replication in the CNS is associated with the acquisition of variants with mutations and deletions within the S protein that alter S dynamics and affinity to the host-cell entry receptor^48,49^. While our studies here were limited to the brain, we hypothesize that tissue-specific patterns of viral diversity may develop across other anatomical sites and contribute to the population structure of the viral quasispecies in an infected host. Indeed, sequencing of SARS-CoV-2 from heart tissue found an overrepresentation of the Spike Q675H mutation, which has been suggested to enhance furin cleavage^50,51^. On the other hand, the immune privileged status of the CNS may facilitate longer infection time courses in those tissues, which would enable a higher degree of genetic diversification specifically in that compartment.

Our data demonstrating trafficking between the CNS and lung suggests the possibility that variants may emerge at distal sites, and may then traffic back to the respiratory tract and spread via respiratory droplets. Over the course of the pandemic, novel variants have emerged that transmit more readily and supplant the dominant circulating strain^9–13^. One source of these variants has been suggested to be immunocompromised hosts, where ongoing viral replication can lead to the emergence of immune escape variants^52–57^. If persistent infection is likewise able to be established in the CNS or in another anatomical compartment, this may serve as an alternate source for population-level variation. Notably, the Spike protein of the Omicron variant has been shown to prefer an endosomal-mediated entry pathway, which would suggest its evolution in a TMPRSS2-low host or site^58^. A better understanding of the viral dynamics in the CNS and other tissues during SARS-CoV-2 infection are required to answer these questions.

Taken together, these data define a selective pressure acting on SARS-CoV-2 Spike during neuroinvasion and compartmental trafficking. Whether direct infection of the CNS is responsible for the neurological complications observed during acute COVID-19 and long-term PASC and how the properties of the virus influence CNS pathology remains unclear. These data may be important for understanding the mechanisms governing intra-host coronavirus evolution, shedding light into the factors that influence the emergence of novel variants and the development of neurological pathologies.

## MATERIALS & METHODS

### Mice and Viral Inoculation.

K18-hACE2 transgenic mice (B6.Cg-Tg(K18-ACE2)2Prlmn/J) mice (Jax Strain # 034860) were purchased from Jackson Laboratoies and were maintained as hemizygotes through breeding at UIC. K18-hACE2 expression was validated through genotyping as described by Jackson Laboratories. Mice had ad libitum access to food and water and kept on a 12-hour light/dark cycle in microisolator cages (Allentown – BCU2) equipped with HEPA filters. BALB/c mice were obtained from Jackson Laboratories (Jax Strain # 00651). For intranasal infection mice were anesthetized with isoflurane and then intranasally inoculated with virus in a 50uL droplet placed on the left and right nostrils of the mouse. Inhalation of the droplet was confirmed for each mouse. For intracranial infection, anesthetized 5-week-old mice were inoculated via direct injection of 1×10^2 PFU of ΔFCS or WA-1 stock in 10uL of PBS through the top of the skull at a depth of 1–2mm with a 29G needle. Mice were euthanized and organs were collected and homogenized in 1ml PBS. Homogenization was achieved with 1mm silicon beads at 5m/s for 60 sec on brain tissue and 3mm silicon beads for 120sec on lung tissue.

### Vaccination.

6–8-week-old K18-hACE2 mice were purchased from Jackson laboratories (Stock No: 034860). Mice were immunized intramuscularly (50 μL per quadriceps) with an Ad5 vector expressing SARS-CoV-2 spike protein (Ad5-S), or nucleocapsid protein (Ad5-N), or both vectors combined; diluted in sterile PBS, at 109 PFU per mouse. Ad5-N was a kind gift of the Masopust/Vezys laboratory^60^.

### Virus Stocks.

SARS-CoV-2, Isolate USA-WA1/2020, NR-52281 was deposited by the Centers for Disease Control and Prevention and obtained through BEI Resources, NIAID, NIH. Virus was propagated on Vero-E6 cells (ATCC). SARS-CoV-2, Mouse-Adapted MA10 Variant infectious clone, NR-55329 was deposited by RS Baric and obtained through BEI Resources, NIAID, NIH. Virus was propagated on Vero-E6 cells (ATCC). SARS-CoV-2 ΔFCS infectious clone and its parent USA-WA1/2020 clone (WA-1) were obtained from the Vineet Menachery Lab (University of Texas Medical Branch). Infectious clone derived viruses were propagated on Vero-E6 cells expressing ACE2 and TMPRSS2. In brief, Vero cells were passaged in DMEM with 10% Fetal bovine serum (FBS) and Glutamax. Cells less than 20 passages were used for all studies. Viral stocks were used after a single expansion (passage = 1) to prevent genetic drift.

### Viral Quantification via Focus Forming Assay.

Focus forming assay (FFA) were performed as described previously^61^. Briefly, serial dilutions of tissue homogenate from inoculated mice were added to a monolayer of Vero-E6 cells in a 96-well plate. One hour after infection, cells were overlaid with 1% (wt/vol) methylcellulose in 2% fetal bovine serum (FBS), 1× minimal essential medium (MEM). 24 h after infection, plates were fixed for 15 min with 4% paraformaldehyde (PFA) followed by 1 hour with 10% neutral buffer formalin (NBF). Staining involved primary antibody polyclonal anti-SARS-CoV-2 guinea pig (BEI Resources – NR10361 1:15000) and secondary antibody goat anti-guinea pig–HRP (Thermo Cat# A16104 1:5000) in PermWash buffer (0.1% saponin, 0.1% BSA, in PBS). Treatment with TrueBlue peroxidase substrate (SeraCare – 5510–0030) produced focus-forming units that were quantified on an ImmunoSpot ELISpot plate scanner (Cellular Technology Limited).

### Viral Quantification by Quantitative PCR.

RNA was isolated using RNeasy Mini Kit (Qiagen Cat# 74104) protocol and eluted in a volume of 40uL of RNase free water. Real-time quantitative reverse transcription PCR was performed using TaqMan 1-step RNA to Ct (Thermo Cat# 4392938) with CDC primer/probe kit (IDT − 10006713) against the N1 gene. Samples were analyzed using Viia7 (ThermoFisher) along with Quantstudio 6 and 7 Flex software (ThermoFisher). Genomes/mL were interpolated using Ct values and genomic standard (BEI - NR-52358) run in triplicate.

### Whole genome sequencing from viral RNA.

cDNA synthesis was performed with SuperScript IV (Thermo 18090200) using random hexamers according to manufacturer’s specifications. Direct amplification of viral genome cDNA was performed as previously described using the Artic Network version 4 primers. Sequencing library preparation of amplicon pools was performed using the SeqWell plexWell 384 kit (Seqwell PW384A) per manufacturer’s instructions. Pooled libraries were sequenced on the Illumina MiSeq using the V2 500 cycle kit. To generate consensus sequences, reads were trimmed to remove adapters and low-quality sequences using Trimmomatic v0.36. Trimmed reads were aligned to the reference genome sequence of SARS-CoV-2 (accession MN908947.3) using bwa v0.7.15. Pileups were generated from the alignment using samtools v1.9 and consensus sequence determined using iVar v1.2.2 with a minimum depth of 10, a minimum base quality score of 20, and a consensus frequency threshold of 0 (i.e., majority base as the consensus).

### Phylogenetic Analysis.

Consensus sequences assembled for each sample were aligned using MAFFT v7.453 software. A Maximum Likelihood (ML) phylogeny with all consensus sequences were inferred with IQ-Tree v2.0.5 using its ModelFinder function before each analysis to estimate the nucleotide substitution model best-fitted for each dataset by means of Bayesian information criterion (BIC). We assessed the tree topology for each phylogeny both with the Shimodaira–Hasegawa approximate likelihood-ratio test (SH-aLRT) and with ultrafast bootstrap (UFboot) with 1000 replicates each. Additionally, with the assembled reads from each sample we performed probabilistic inference of intra-host viral quasispecies of the Spike gene for each sample using QuasiRecomb. The sequences of the inferred viral haplotypes from each quasispecies were also aligned and ML phylogenies inferred using the same approach as the consensus analysis. All final tree representation was performed with the R package ggtree v3.2.1.

### Viral Diversity Analysis.

To study and compare intra-host diversification in different animals and tissues, Shannon Entropy was calculated using the nucleotide frequencies obtained from iVar applying the formula: Sh = SUM[−(pi)·log2(pi)]; where Sh is Shannon Entropy calculated for each position and pi is the frequency of each nucleotide in each position. To ensure a robust estimation of diversity, Shannon Entropy calculations were limited to positions with a minimum read depth of 100 reads to ensure robustness of the measurement. To test for significant differences in overall genetic entropy between compartments, we used the genetic entropy values for each nucleotide position in every animal and tissue and fitted a log-transformed linear mixed effects model, using animal and nucleotide position as random effects. For these analysis we used lme4 version 1.1–34 in R version 4.0.3.

### Pseudovirus Generation and Entry Assay.

Pseudoviruses were created using plasmids for SARS-CoV-2 WA1/2020 spike, SARS-CoV-2 ΔFCS and HIV-1 proviral vector pNL4–3.Luc.R-E-(from the NIH AIDS Research and Reference Reagent Program) containing a luciferase reporter gene. Pseudovirions were created following a polyethylenimine (PEI)-based transient co-transfection on 293T cells. After 5 h, cells were washed with PBS and the medium was replaced with phenol red-free DMEM. 16 h post-transfection, supernatants were collected and filtered through 0.45 μm pore size filters. Low passage VeroE6-ACE2-TMPRSS2 (VAT) cells were seeded in 96-well plates at the density of 5000 cell/well and incubated at 37°C and 5% CO_2_ for 24 h before infection. In the presence of 10uM concentration of Aloxostatin (MedChem Express – HY-100229) or Camostat mesylate (MedChem Express – HY-13512), VAT cells were infected with pseudovirions containing a luciferase reporter gene. All drugs were dissolved in dimethyl sulfoxide (DMSO) and final treatment DMSO concentrations of 1%. Plates were incubated at 37°C and 5% CO_2_ for 48 h and viral infection was determined by luminescence using the neolite reporter gene assay system (PerkinElmer − 6016716). Virus with 1% DMSO was used as a negative control and data were normalized to the negative control.

### Western blot.

To isolate protein, viral stock or pseudovirus were prepared using 4x Laemmli sample buffer (Thermo – NP0007) and reducing agent (Thermo – NP0004). Samples were inactivated and denatured by 70°C heat for 10 minutes and loaded onto a 4–12% Bi-Tris gel (Thermo – NP0321BOX) submerged in 1x MEM SDS Running buffer (Thermo – NP0002). Protein was transferred to polyvinylidene difluoride (PVDF) membrane and stained with 1:2000 dilution of polyclonal anti-SARS guinea pig antibody (BEI Resources - NR-10361) followed by probing with 1:10000 HRP-conjugated anti-rabbit antibody (Invitrogen – 65–6120). Signal was developed by treating membranes with Clarity Western ECL Substrate (Bio-Rad – 170–5060) and imaged on a ChemiDoc MP Imaging System (Bio-Rad).

### Infection with Lung Homogenate.

Lung Homogenate was added to a monolayer of VeroE6-ACE2-TMPRSS2 cells in 12 well plate. Cells were infected for 1 hour at 37°C, washed with PBS, then given fresh DMEM with 10% FBS and GlutaMax. For UV inactivation, 500uL of homogenate was placed in 10mm dish and exposed to direct UV light for 1 hour at room temperature.
